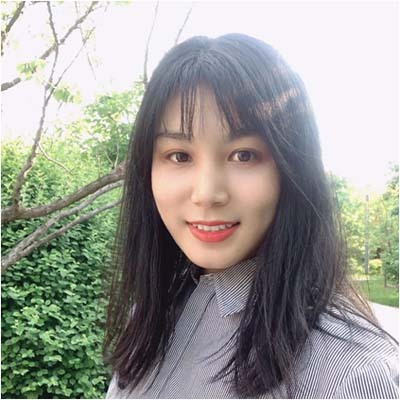# Light People: Professor Aydogan Ozcan

**DOI:** 10.1038/s41377-021-00643-1

**Published:** 2021-10-05

**Authors:** Tingting Sun

**Affiliations:** Light Publishing Group, Changchun Institute of Optics, Fine Mechanics and Physics, Chinese Academy of Sciences, 3888 Dong Nan Hu Road, Changchun, 130033 China

**Keywords:** Imaging and sensing, Microscopy

## Abstract

In 2016, the news that Google’s artificial intelligence (AI) robot AlphaGo, based on the principle of deep learning, won the victory over lee Sedol, the former world Go champion and the famous 9th Dan competitor of Korea, caused a sensation in both fields of AI and Go, which brought epoch-making significance to the development of deep learning. Deep learning is a complex machine learning algorithm that uses multiple layers of artificial neural networks to automatically analyze signals or data. At present, deep learning has penetrated into our daily life, such as the applications of face recognition and speech recognition. Scientists have also made many remarkable achievements based on deep learning. Professor Aydogan Ozcan from the University of California, Los Angeles (UCLA) led his team to research deep learning algorithms, which provided new ideas for the exploring of optical computational imaging and sensing technology, and introduced image generation and reconstruction methods which brought major technological innovations to the development of related fields. Optical designs and devices are moving from being physically driven to being data-driven. We are much honored to have Aydogan Ozcan, Fellow of the National Academy of Inventors and Chancellor’s Professor of UCLA, to unscramble his latest scientific research results and foresight for the future development of related fields, and to share his journey of pursuing Optics, his indissoluble relationship with Light: Science & Applications (LSA), and his experience in talent cultivation.

## Abstract (web summary)

A word leading scientist in optical computational imaging and sensing, Prof. Aydogan Ozcan mirrored the ideal into reality with unremitting explorations.

## Graphical abstract

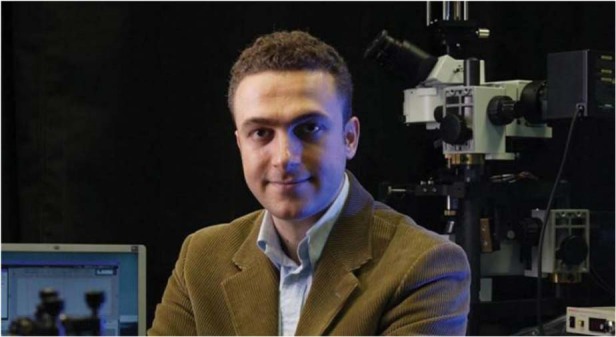

**Figure:**
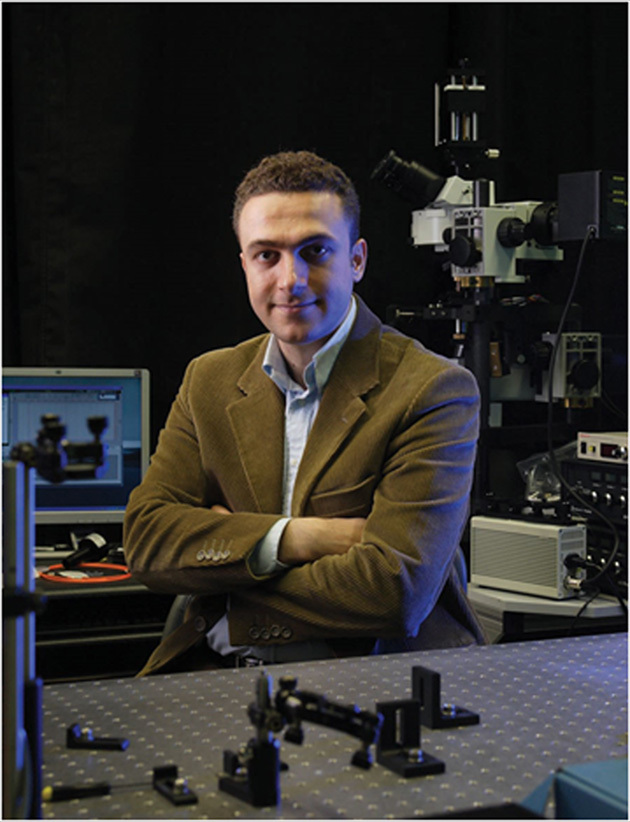

**Biography:** Prof. Aydogan Ozcan is the Chancellor’s Professor and the Volgenau Chair for Engineering Innovation at UCLA and an HHMI Professor with the Howard Hughes Medical Institute, leading the Bio- and Nano-Photonics Laboratory at UCLA School of Engineering and is also the Associate Director of the California NanoSystems Institute. Dr. Ozcan is elected Fellow of the National Academy of Inventors (NAI) and holds >45 issued/granted patents and >20 pending patent applications and is also the author of one book and the co-author of >700 peer-reviewed publications in major scientific journals and conferences. Dr. Ozcan is the founder and a member of the Board of Directors of Lucendi Inc., Hana Diagnostics, Pictor Labs, as well as Holomic/Cellmic LLC, which was named a Technology Pioneer by The World Economic Forum in 2015. Dr. Ozcan is also a Fellow of the American Association for the Advancement of Science (AAAS), the International Photonics Society (SPIE), the Optical Society of America (OSA), the American Institute for Medical and Biological Engineering (AIMBE), the Institute of Electrical and Electronics Engineers (IEEE), the Royal Society of Chemistry (RSC), the American Physical Society (APS) and the Guggenheim Foundation, and has received major awards including the Presidential Early Career Award for Scientists and Engineers, International Commission for Optics Prize, Biophotonics Technology Innovator Award, Rahmi M. Koc Science Medal, International Photonics Society Early Career Achievement Award, Army Young Investigator Award, NSF CAREER Award, NIH Director’s New Innovator Award, Navy Young Investigator Award, IEEE Photonics Society Young Investigator Award and Distinguished Lecturer Award, National Geographic Emerging Explorer Award, National Academy of Engineering The Grainger Foundation Frontiers of Engineering Award and MIT’s TR35 Award for his seminal contributions to computational imaging, sensing and diagnostics. Dr. Ozcan is also listed as a Highly Cited Researcher by Web of Science, Clarivate, and serves as the co-Editor-in-Chief of eLight.

**Q1: Can you please briefly define with your own words the focus of your research lab?**

A1: My lab is composed of three sub-areas:

- Computational microscopy which includes e.g., deep learning-enabled microscopy, holography, lensless imaging, on-chip microscopy, 3D microscopy, imaging flow-cytometry, among others.
- Sensing: point-of-care sensors, mobile-phone enabled sensors, field-based sensing and measurement systems with applications in mobile health and telemedicine, environmental monitoring (for example, air/water quality sensing).
- Optical computing and inverse design: diffractive networks, diffractive optical processors, and deep learning-designed free-space optics.

While conducting exciting, cutting edge applied research on photonics and optics, we are also training the next generation of engineers, scientists and entrepreneurs through our research programs. Some of our trainees have started up their own labs in the United States, China and other parts of the world, some went to industry, leading their own teams, and some started up companies.

**Q2: Optical coherence tomography (OCT) is a widely used imaging method that provides three-dimensional information about the optical scattering properties of biological samples. One of your recent publications in LSA focused on improving image reconstruction in OCT** ^1^ **. Could you please introduce the research idea and main advantages of your method as well as its impact for OCT field?**

A2: Indeed, OCT is widely used in diagnostic medicine, for example in ophthalmology, to noninvasively obtain detailed 3D images of the retina and underlying tissue structure. In our latest paper^1^ published in LSA, we have developed a deep learning-based OCT image reconstruction method that can successfully generate 3D images of tissue specimen using significantly less spectral data than normally required. Using standard image reconstruction methods employed in OCT, undersampled spectral data, where some of the spectral measurements are omitted, result in severe spatial artifacts in the reconstructed images, obscuring 3D information and structural details of the sample to be visualized. In our new approach, we trained a neural network using deep learning to rapidly reconstruct 3D images of tissue samples with much less spectral data than normally acquired in a typical OCT system, successfully removing the spatial artifacts observed in standard image reconstruction methods. The efficacy and robustness of this new method was demonstrated by imaging various human and mouse tissue samples using threefold less spectral data captured by a state-of-the-art swept-source OCT system. Running on graphics processing units (GPUs), the neural network successfully eliminated severe spatial artifacts due to undersampling and omission of most spectral data points in less than 1 ms for an OCT image that is composed of 512 depth scans (A-lines). These results that we have recently published in LSA highlight the transformative potential of this neural network-based OCT image reconstruction framework, which can be easily integrated with various spectral domain OCT systems, to improve their 3D imaging speed without sacrificing resolution or signal-to-noise of the reconstructed images.

**Figure:**
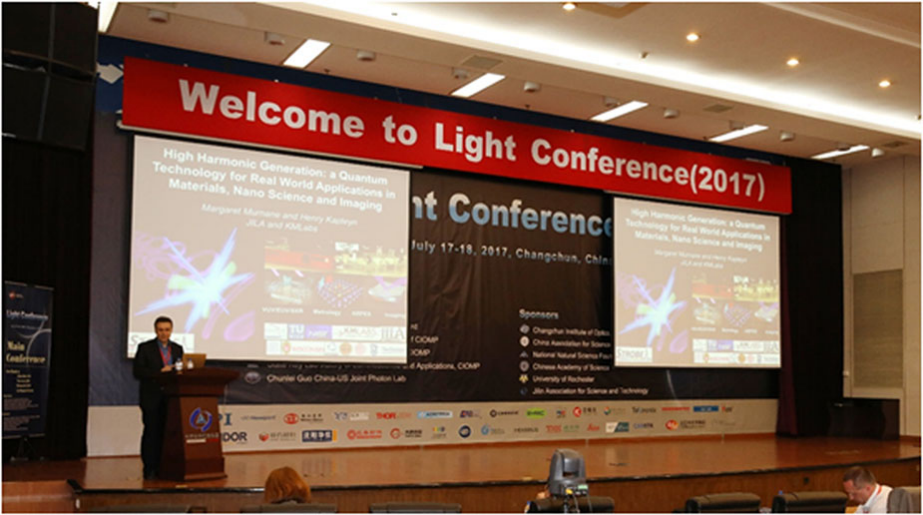
Prof Ozcan presenting at 2017 Light Conference.

**Q3: In 2017, your paper “Phase Recovery and Holographic Image Reconstruction using Deep Learning in Neural Networks** ^2^ **” published in LSA has got 359 citations on the Web of Science, and also won the LSA annual Outstanding Paper, Highly Cited Paper, Popular Downloaded Paper, which became a major breakthrough in the field of optical computational imaging. Can you talk about the development status and future trends of computational imaging? What are the potential applications?**

A3: This seminal LSA paper of our team was published online in October 2017, and it was placed in arXiv by our team in May 2017. These results demonstrate the first use of deep neural networks for holographic image reconstruction and phase recovery. In this paper, we demonstrated that a convolutional neural network (CNN) can learn to perform phase recovery and holographic image reconstruction after appropriate training. This deep learning-based approach provides a fundamentally new framework to conduct holographic imaging by rapidly eliminating twin-image and self-interference related spatial artifacts, which have been part of holography since its invention by Dennis Gabor (leading to the Nobel Prize in Physics, 1971). Compared to existing holographic phase recovery approaches, this neural network framework is significantly faster to compute and reconstructs much improved phase and amplitude images of the objects using a single hologram, i.e., requires less number of measurements in addition to being computationally faster. Remarkably, this deep learning based twin-image elimination and phase recovery have been achieved without any modeling of light-matter interaction or a solution of the wave equation.

After this 2017 LSA publication, our team has further expanded these results in many unique ways. In an Optica paper^3^ published in 2018, we demonstrated an innovative application of deep learning to significantly extend the imaging depth of a hologram. We demonstrated a CNN-based approach that simultaneously performs auto-focusing and phase-recovery to significantly extend the depth-of-field (DOF) in holographic image reconstruction. For this, a CNN is trained by using pairs of randomly de-focused back-propagated holograms and their corresponding in-focus phase-recovered images. After this training phase, the CNN takes a single back-propagated hologram of a 3D sample as input to rapidly achieve phase-recovery and reconstruct an in focus image of the sample over a significantly extended DOF. Furthermore, this deep learning based auto-focusing and phase-recovery method is non-iterative, and significantly improves the algorithm time-complexity of holographic image reconstruction from O(*nm*) to O(1), where *n* refers to the number of individual object points or particles within the sample volume, and *m* represents the discrete focusing search space, within which each object point or particle needs to be individually focused.

Another breakthrough result, in my opinion, that is at the intersection of deep learning and holography was published in LSA in 2019, and this work was placed in arXiv in 2018 by our team. In this publication^4^, we introduced the use of a deep neural network to perform cross-modality image transformation from a digitally back-propagated hologram corresponding to a given depth within the sample volume into an image that is equivalent to a bright-field microscope image acquired at the same depth. Because a single hologram is used to digitally propagate to different sections of the sample to virtually generate bright-field equivalent images of each section, this approach bridges the volumetric imaging capability of digital holography with speckle- and artifact-free image contrast of bright-field microscopy. After its training, the deep neural network learns the statistical image transformation between a holographic imaging system and an incoherent bright-field microscope, and intuitively it brings together “the best of both worlds” by fusing the advantages of holographic and incoherent bright-field imaging modalities.

For this holographic to bright-field image transformation, we used a generative adversarial network (GAN), which was trained using pollen samples imaged by an in-line holographic microscope along with a bright-field incoherent microscope (used as the ground truth). After the training phase, which needs to be performed only once, the generator network blindly takes as input a new hologram (never seen by the network before) to infer its bright-field equivalent image at any arbitrary depth within the sample volume. We experimentally demonstrated the success of this powerful cross-modality image transformation between holography and bright-field microscopy, where the network output images were free from speckle and various other interferometric artifacts observed in holography, matching the contrast and DOF of bright-field microscopy images that were mechanically focused onto the same planes within the 3D sample. We also demonstrated that the deep network also correctly colorizes the output image, using an input hologram acquired with a monochrome sensor and narrow-band illumination, matching the color distribution of the bright-field image.

This deep learning-enabled image transformation between holography and bright-field microscopy replaces the need to mechanically scan a volumetric sample, as it benefits from the digital wave-propagation framework of holography to virtually scan through the sample, where each one of these digitally propagated fields are transformed into bright-field microscopy equivalent images, exhibiting the spatial and color contrast as well as the DOF expected from an incoherent microscope.

**Figure:**
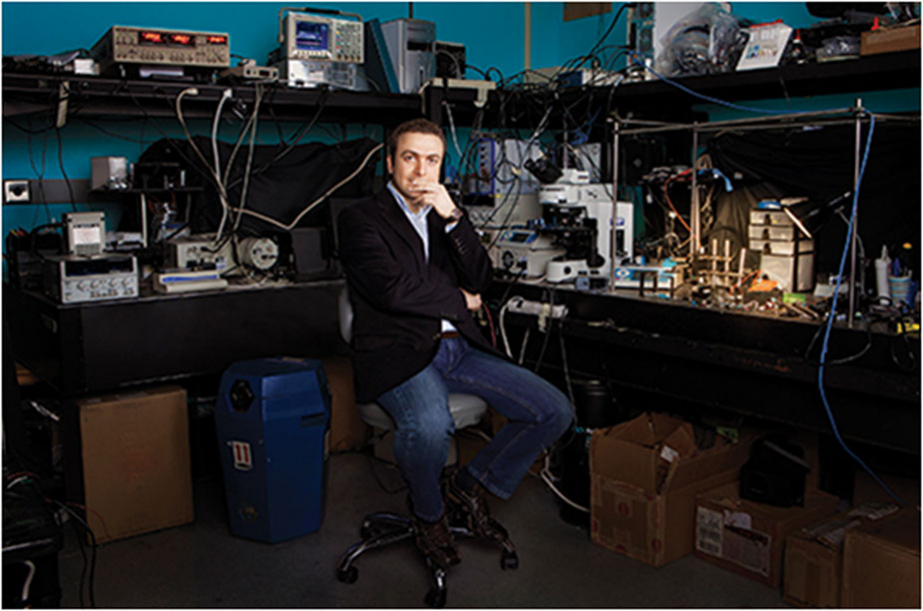
Prof. Aydogan Ozcan in the lab.

**Q4: You lead your team to research Diffraction Deep Neural Network and moved the Neural Network from the chip to the real world, which realized deep learning with almost zero energy consumption and zero delay by relying on the light propagation. The results were also successfully published in Science** ^5^ **. Can you talk about the subtlety of this research work? For the further development of this technology, what are the key problems to be solved currently?**

A4: Diffractive Optical Networks have been introduced by my lab as a machine learning framework that unifies deep learning-based training of matter with the physical models governing the light propagation and diffraction to enable optical inference through a set of diffractive layers (published in Science, 2018)^5^. The forward model of a diffractive optical network can be mathematically formulated as a complex-valued matrix operator that multiplies an input field to create an output field at the detector plane/aperture. This operator is designed/trained using deep learning to transform a set of complex fields (forming, e.g., the object data classes) at the input aperture of the optical network into another set of corresponding fields at the output aperture (forming, e.g., the data classification signals) and is physically created through the interaction of the input light with the designed diffractive surfaces as well as free-space propagation within the network. The training stage of a diffractive network is performed using a computer, and relies on deep learning and iterative error backpropagation methods to tailor the light-matter interaction across a set of diffractive layers that collectively perform a given machine learning task, such as object classification. After this numerical training phase implemented in a computer, the diffractive network design is fixed and the transmission or reflection coefficients of the neurons of all the layers are determined. This diffractive design, once physically fabricated using, e.g., 3D-printing, lithography, etc., can then perform at the speed of light, the specific task that it is trained for, using only optical diffraction and passive optical layers, creating an efficient and fast way of implementing optical inference.

Our previous studies on diffractive optical networks have demonstrated the generalization capability of these multi-layer diffractive designs to new, unseen image data. For example, using a 5-layer diffractive network architecture, all-optical blind testing accuracies of >98% and >90% have been reported for the classification of the images of handwritten digits (MNIST dataset) and fashion objects (Fashion-MNIST dataset) that were encoded in the amplitude and phase channels of the input object plane, respectively. For more complex and much harder to classify image datasets such as CIFAR-10, a substantial improvement in the inference performance of diffractive networks was demonstrated using ensemble learning^6^ published in LSA in 2021. After independently training >1250 individual diffractive networks that were diversely designed with a variety of passive input filters, a pruning algorithm was applied to select an optimized ensemble of diffractive networks that collectively improved the image classification accuracy. Through this pruning strategy, an ensemble of *N* = 14 diffractive networks collectively achieved a blind testing accuracy of 61.14% on the classification of CIFAR-10 test images, providing an inference improvement of 16.6% compared to the average performance of the individual diffractive networks within each ensemble, demonstrating the “wisdom of the crowd”.

Successful experimental demonstrations of these all-optical image classification systems have been reported using 3D-printed diffractive layers that conduct inference by modulating the in-coming object wave at terahertz (THz) wavelengths. In addition to statistical inference, the same optical information processing framework of diffractive networks has also been utilized to design deterministic optical components, for e.g., ultra-short pulse shaping^7^, spectral filtering and wavelength division multiplexing^8^. In these latter examples, the input field was known a priori and was fixed, i.e., unchanged, where the task of the diffractive network was to perform a deterministic, desired transformation for a given/known optical input.

Despite the lack of nonlinear optical elements in these previous implementations, diffractive optical networks have been shown to offer significant advantages in terms of (1) inference/classification accuracy, (2) diffraction efficiency, and (3) output signal contrast, when the number of successive diffractive layers in the network design is increased. We published an important work in LSA detailing some of these characteristics of diffractive optical networks, which is titled: “All-Optical Information Processing Capacity of Diffractive Surfaces^9^”.

Diffractive optical networks have also been extended to harness broadband input radiation to design spectrally encoded single-pixel machine vision systems, where unknown input objects were classified and reconstructed through a single-pixel detector^10^. This single-pixel machine vision framework achieved >96% blind testing accuracy for optical classification of handwritten digits (MNIST dataset) based on the spectral power detected at ten distinct wavelengths, each assigned to one digit/class. In addition to the optical classification of objects through spectral encoding of data classes, a shallow electronic neural network with two hidden layers was trained (after the diffractive network training) to rapidly reconstruct the images of the classified objects based on their diffracted power spectra detected by a single-pixel spectroscopic detector. Using only 10 inputs, one for each wavelength, this shallow network successfully reconstructed the images of the input, unknown objects, even if they were (rarely) incorrectly classified by the trained diffractive network. Considering the fact that each image of a handwritten digit is composed of ~800 pixels, this shallow image reconstruction network, with an input vector size of 10, performs a form of image decompression to successfully decode the task-specific spectral encoding of the diffractive network.

In one of our latest papers published in LSA^11^, we also report the design of diffractive surfaces to all-optically perform arbitrary complex-valued linear transformations between an input and output field-of-view. Stated differently, we demonstrated in this recent work the universal approximation capability of diffractive networks for all-optically synthesizing any arbitrarily selected linear transformation with independent phase and amplitude channels. Our methods and conclusions in this recent LSA work can be broadly applied to any part of the electromagnetic spectrum to design all-optical processors using spatially engineered diffractive surfaces to universally perform an arbitrary complex-valued linear transformation. Therefore, these results have wide implications and can be used to design and investigate various coherent optical processors formed by diffractive surfaces such as metamaterials, plasmonic or dielectric-based metasurfaces, as well as flat optics-based designer surfaces that can form information processing networks to execute a desired computational task between an input and output aperture.

**Figure:**
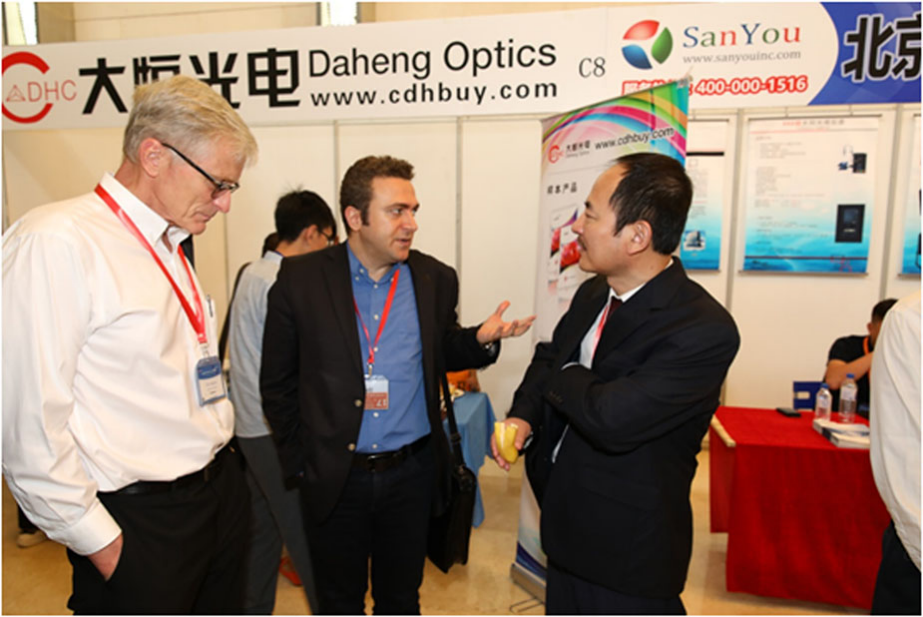
Prof. Ozcan communicating with Prof. Olav Solgaard (left) and Prof. Tianhong Cui (right) at 2017 Light Conference in Changchun.

**Q5: Deep learning is a class of machine learning techniques that use multiple layers of artificial neural networks to automatically analyze signals or data. The introduction of deep learning brings new opportunities for the development of optical microscopy. Can you talk about the current status of optical microscopy? What revolutions has deep learning brought to optical microscopy technique? What are the application prospects?**

A5: In addition to holography, phase recovery and holographic image reconstruction that I described earlier, another area that our lab has made seminal contributions to is deep learning microscopy. In fact, the first use of deep neural networks for advancing the resolution, field-of-view and DOF of optical microscopy has been demonstrated by our team in 2017. This work of our lab titled “Deep Learning Microscopy^12^” was published in Optica in November 2017 and was put in arXiv by our team in May 2017, receiving so far >400 citations. This seminal work was followed by many research labs, bringing in new computational tools to microscopy field, powered by deep learning.

In a follow-up work by our team, we published another very important result^13^ in Nature Methods demonstrating super-resolution imaging in fluorescence microscopy and beating the diffraction limit of light through cross-modality image transformations from e.g. confocal microscopy to STED or TIRF to SIM. Perhaps one of the most surprising results that we have got on this line of research was on 3D virtual refocusing of fluorescence microscopy images using deep learning^14^, also published in Nature Methods. In this work, we introduced a new framework (termed Deep-Z) that is enabled by deep learning to statistically learn and harness hidden spatial features in a fluorescence image to virtually propagate a single fluorescence image onto user-defined 3D surfaces within a fluorescent sample volume. This is achieved without any mechanical scanning, additional optical hardware/components or a trade-off of imaging resolution, field-of-view or speed. Stated differently, we introduced, for the first time, a digital propagation framework that learns (through only image data and without any assumptions or theoretical models) the spatial features in fluorescence images that uniquely encode the 3D fluorescence wave propagation information in an intensity-only 2D recording without additional hardware.

There are various powerful features of Deep-Z that make it transformative for fluorescence imaging at all scales and across various disciplines that use fluorescence. A unique capability of Deep-Z framework is that it enables digital propagation of a fluorescence image of a 3D surface onto another 3D surface, user-defined by the pixel mapping of the corresponding digital propagation matrix (DPM). An analog of the same capability exists in holography under the paraxial approximation. In this sense, Deep-Z framework brings the computational 3D image propagation advantage of holography or other coherent imaging modalities into fluorescence microscopy. Such a unique capability can be useful, among many applications, for e.g., simultaneous auto-focusing of different parts of a fluorescence image after the image capture, measurement or assessment of aberrations introduced by the optical system as well as for correction of such aberrations by applying a desired nonuniform DPM. To exemplify the power of this additional degree of freedom enabled by Deep-Z, we experimentally demonstrated the correction of the planar tilting and curvature of different samples after the acquisition of a single 2D fluorescence image per object.

Yet another unique feature of this Deep-Z framework is that it permits cross-modality digital propagation of fluorescence images, where the neural network is trained with gold standard label images obtained by a different fluorescence microscopy modality to teach the generator network to digitally propagate an input image onto another plane within the sample volume, but this time to match the image of the same plane that is acquired by a different fluorescence imaging modality compared to the input image. We term this related framework Deep-Z+. To demonstrate the proof of concept of this unique capability, we trained Deep-Z+ with input and label images that were acquired with a wide-field fluorescence microscope and a confocal microscope, respectively, to blindly generate at the output of this cross-modality network, digitally propagated images of an input fluorescence image that match confocal microscopy images of the same sample sections, i.e., performing axial sectioning (similar to the contrast that a confocal microscope has) and digital propagation of a fluorescence image, both at the same time.

After its training, Deep-Z remains fixed, and its non-iterative inference requires no parameter estimation or search. The inference is performed in a rapid fashion, as it outputs the desired digitally-propagated fluorescence image without any changes to the optical microscopy set-up, or a trade-off of its spatial resolution or field-of-view. As such, it allows rapid volumetric imaging (limited only by the detector speed) without any axial scanning or hardware modifications. In addition to fluorescence microscopy, Deep-Z framework might be applied to other incoherent imaging modalities, and in fact it provides a bridge to close the gap between coherent and incoherent microscopes by enabling computational 3D imaging of a volume using a single 2D incoherent image.

**Q6: Prior to joining UCLA in 2007, you received your Ph.D. and completed postdoctoral fellowship at Stanford University and were appointed as a research faculty at Harvard Medical School, Wellman Center for Photomedicine. How did this experience influence your subsequent scientific career? Why did you later consider to join UCLA?**

A6: After an engineering PhD at Stanford, moving to a medical school to conduct research was an eye opening experience for me. At Harvard Medical School, I learned how an MD looks at innovation from the perspective of impact—not novelty. An engineer, sometimes, can get obsessed with novelty and “coolness” of an invention or idea—but biomedical research first and foremost cares about impact, especially from the perspective of improving human health. My experience at Stanford and Harvard Medical School (Wellman Center for Photomedicine) taught me the importance of the intersection set between Impact and Novelty. That shaped how I look at science and engineering from the perspective of “innovations that matter”. Gary Tearney and Brett Bouma, pioneers in OCT field have been my mentors at Harvard and I would like to acknowledge them for their contributions to my training.

**Figure:**
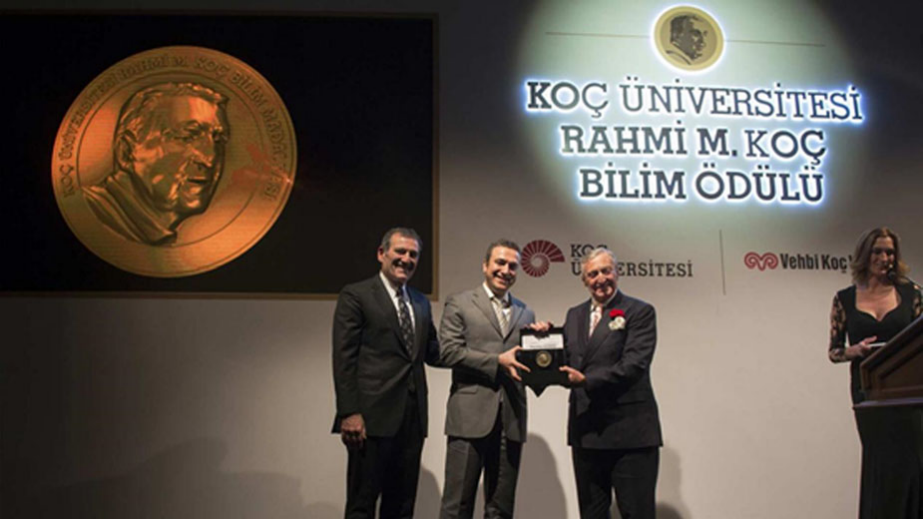
Prof. Ozcan receiving the Rahmi M. Koc Science Medal from Koc University and Vehbi Koc Foundation. This Science Medal carried the name of Rahmi M. Koc, one of the most prestigious businessman and philanthropist in Turkey. Prof. Ozcan was inaugural recipient of this prestigious award in 2016 and received it in Istanbul right after his 38th birthday.

**Q7: You’ve been working on optics for many years and have become a world lead scientist in related fields, so we are wondering, what are your plans for your future development of scientific research?**

A7: A very exciting development in my career right now is a new spin-off company from my lab that aims to transform a century-old field and redefine histopathology and how it is practiced: Pictor Labs: https://www.pictor-labs.com/

Approximately, 2 years ago, my lab published a paper in Nature Biomedical Engineering which introduced a deep learning-based method to “virtually stain” autofluorescence images of unlabeled histological tissue sections, eliminating the need for chemical staining^15^. This technology was developed to leverage the speed and computational power of deep learning to improve upon century-old histochemical staining techniques which can be slow, laborious and expensive. In this seminal paper we showed that this virtual staining technology using deep neural networks is capable of generating highly accurate stains across a wide variety of tissue and stain types. It has the potential to revolutionize the field of histopathology by reducing the cost of tissue staining, while making it much faster, less destructive to the tissue and more consistent/repeatable.

Since the publication of our paper, we have had a number of exciting developments moving the technology forward. We have continued to find new applications for this unique technology, using the computational nature of the technique to generate stains which would be impossible to create using traditional histochemical staining. For example, we have used of what we refer to as a “digital staining matrix” which allows us to generate and digitally blend multiple stains using a single deep neural network, by specifying which stain should be performed on the pixel level. Not only can this unique framework be used to perform multiple different stains on a single tissue section, it can also be used to create micro-structured stains, digitally staining different areas of labelfree tissue with different stains. Furthermore, this digital staining matrix enables these stains to blended together, by setting the encoding matrix to be a mixture of the possible stains. This technology can be used to ensure that pathologists are able to receive the most relevant information possible from the various virtual stains being performed. This work^16^ was published in LSA in 2020 and has opened up the path for a very exciting new opportunity: stain-to-stain transformations, which enable transforming existing images of tissue biopsy stained with one type of stain into many other types of stains^17^, almost instantaneously. Published in Nature Communications, this stain-to-stain transformation process takes less than one minute per tissue sample, as opposed to several hours or even more than a day when performed by human experts. And, this speed differential enables faster preliminary diagnoses that require special stains, while also providing significant savings in costs.

Motivated by transformative potential of our virtual staining technology, we have also begun the process of its commercialization and founded Pictor Labs, a new Los Angeles-based startup. Pictor in Latin means “painter” and at Pictor Labs we virtually “paint” the microstructure of tissue samples using deep learning. In 2020, we were successful in raising seed funding from venture capital firms including M Ventures (a subsidy of Merck KGaA), Motus Ventures, as well as private investors.

Through Pictor labs, we aim to revolutionize the histopathology staining workflow using this virtual staining technology, and by building a cloud computing-based platform which facilitates histopathology through AI, we will enable tissue diagnoses and help clinicians manage patient care. I am very excited to have this unique opportunity to bring our cutting-edge academic research into the commercialization phase and look forward to more directly impacting human health over the coming years using this transformative virtual staining technology.

**Figure:**
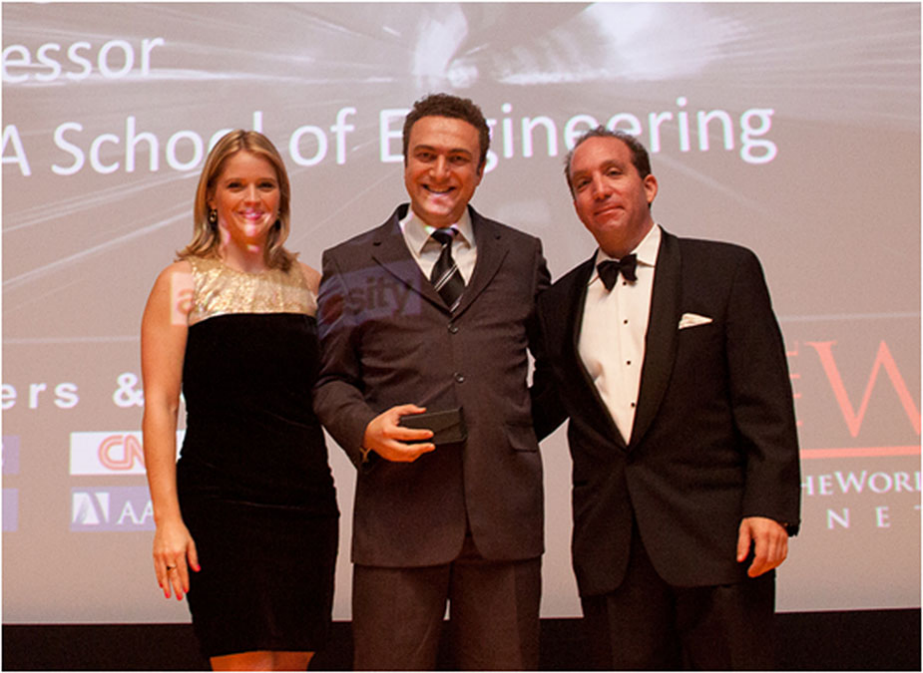
Prof. Ozcan receiving the World Technology Award on Health & Medicine, presented by the World Technology Network in association with TIME, CNN, AAAS, Science, Technology Review, Fortune and Kurzweil (2012).

**Q8: The scientific research should finally serve to improve people’s livelihood and economic development, and the transformation from academic to industrial is an important process. At present, which ones of your research works have realized achievement transformation? What are the social benefits?**

A8: My research on computational imaging, mobile sensing and diagnostics created widely scalable mobile technologies, for e.g., blood analysis, sensing of pathogens/toxins in bodily fluids, food and water samples, diagnosis of infectious diseases, screening of antimicrobial resistance, pathology analysis, as well as particulate matter and bio-aerosol detection for air quality measurements, which altogether have the potential to dramatically increase the reach of advanced biomedical technologies to developing countries and resource limited settings. My work has been broadly helping to democratize biomedical measurement science by enabling advanced measurements to be cost-effectively performed even in field-settings using mobile instruments powered by computational optics and machine learning.

These innovative measurement technologies led to >45 issued patents, several licensed by different companies including Honeywell, GE, Northrup Grumman (Litton), Arcelik, NOW Diagnostics and my own start-ups, targeting multi-billion $ markets, with products used in >10 countries, also earning one of my own companies a “Technology Pioneer” Award given by The World Economic Forum in 2015.

My work on mobile microscopy entered introductory level biology textbooks by, e.g., National Geographic, Cengage, and is being used by numerous academic groups world-wide, including in developing countries, also through my lab’s massive collaborations with >25 labs.

My lab was one of the first teams that utilized the cellphone as a platform for advanced measurements, microscopy and sensing covering various applications. For example, we were the first group to image and count individual viruses, individual DNA molecules using mobile phone-based microscopes. We were one of the first groups to utilize the smart phone as a platform for quantitative sensing, for example, quantification of lateral flow tests. Our mobile diagnostic test readers are still being used in industry through a licensee of one of our patents. Lucendi, a start-up that I cofounded, has commercialized a mobile imaging flow cytometer for water quality analysis, including for example alga toxic blooms. In fact this seminal work^18^ was also published in LSA.

As another example, our team has introduced the first point of care sensor that is designed by machine learning and that runs and makes decisions based on a neural network^19,20^. This is a vertical flow assay that can look at >80 immunoreactions in parallel. We have shown its efficacy for detection of early stage Lyme disease patients based on IgG and IgM panels (profiling the immunity of the patient), published in ACS Nano. We are also considering a similar approach for COVID-19—especially important to understand, e.g., the efficacy of vaccines and when a booster shot is needed.

**Figure:**
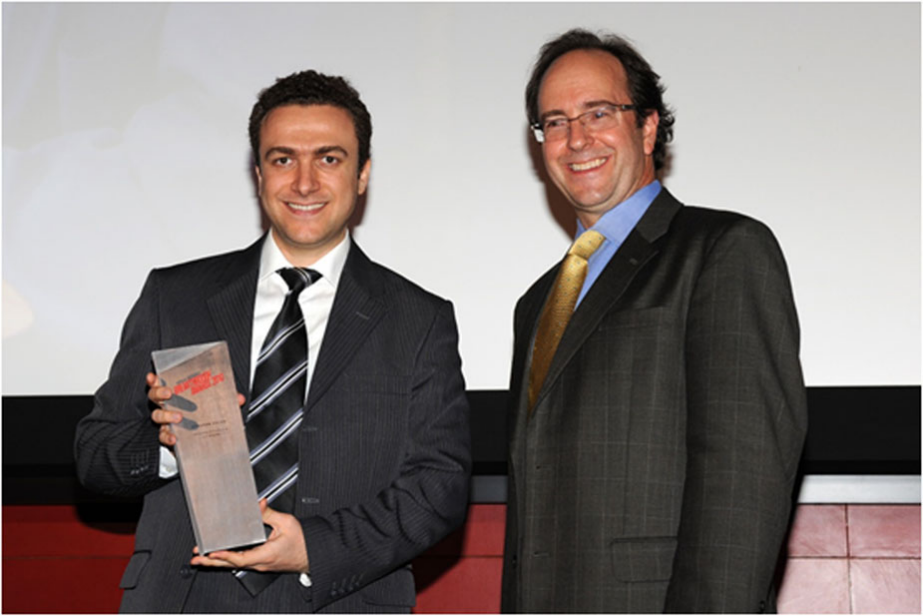
Prof. Ozcan receiving the Popular Mechanics Breakthrough Award.

**Figure:**
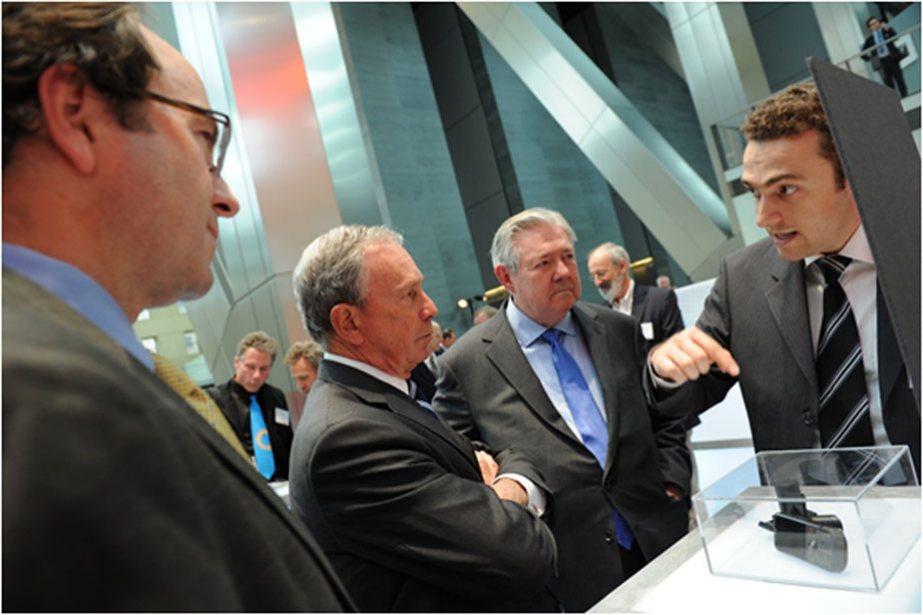
Prof. Ozcan explaining his mobile microscopy technology to Michael Bloomberg.

**Q9: We officially launched eLight journal in June this year, which aims to attract the finest manuscripts, broadly covering all sub-fields of optics, photonics and electromagnetics, in particular focusing on those emerging topics and cross-disciplinary researches related to optics. As the Editor-in-Chief, can you talk about the development orientation and direction of eLight? How can this new journal stand out from its peers? What is your vision and expectation for eLight?**

A9: eLight is the new kid in the block. Together with Cheng-Wei, I am serving as the co-EIC of eLight and I am also the father of its name. LSA is an amazing success story in Optics/Photonics field, with an impact factor of 17.782—it is a global leading journal in optics, and CIOMP should be proud of it. eLight will be powered by the same team that has made LSA an amazing success. Approximately, 50 publications will be targeted per year. So my message to all the researchers who are focusing on Optics/Photonics field, broadly defined:

Think of your best work in a given year—that would provide an excellent fit to eLight. Please consider submitting your best work to eLight.

**Figure:**
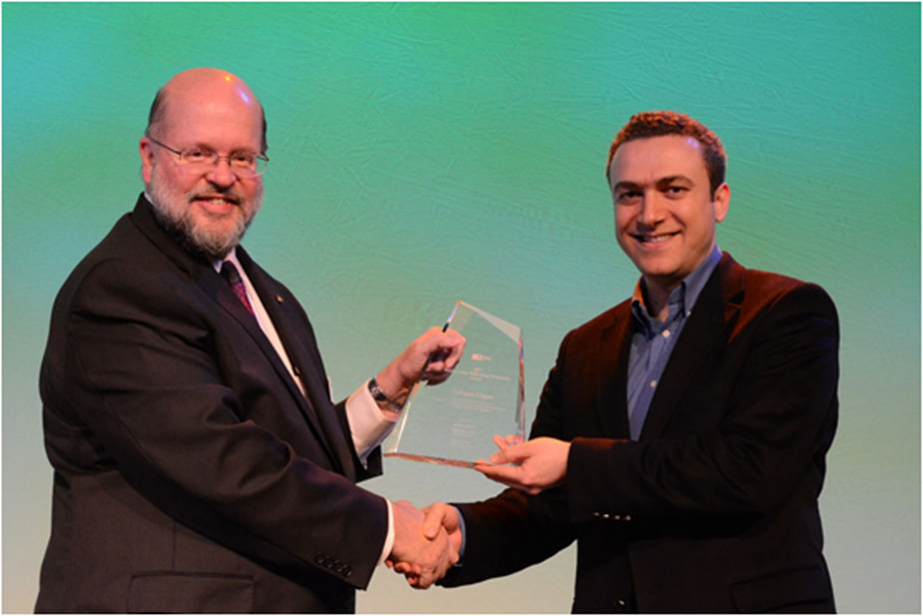
Prof. Ozcan is the inaugural recipient of the SPIE Biophotonics Technology Innovator Award.

**Q10: In our impression, you are working almost all the time, and it seems that whenever send you a message, we can get a response at once. We really admire your professionalism a lot. How do you maintain your robust energy and positive attitude at work? In your opinion, what characters should an excellent scientific researcher possess?**

A10: You have got to constantly learn new things. My lab is not focusing on a narrow area, and effectively it has three different labs come together. This gives us the big picture and the ability to connect things, because we can see how different areas can come together by looking at the big picture. This also makes it much bigger than the sum of its parts.

Running a large engineering lab with a broad focus is a lot of effort, but to succeed in all of these areas you need to build interdisciplinary teams with a culture of sharing and learning from each other. My team is very diverse, covering many areas of engineering, and everyone can learn from everyone else. At the same time, we have many interdisciplinary collaborators. This makes our publications high impact and relevant—meaning, we solve problems that really matter—at the intersection of impact and novelty.

In summary: Never stop learning. Be open minded to new directions. Learn from your students, team members and colleagues. And understand that everyone can be wrong, including yourself. No one is perfect and understanding your weaknesses is important.

**Figure:**
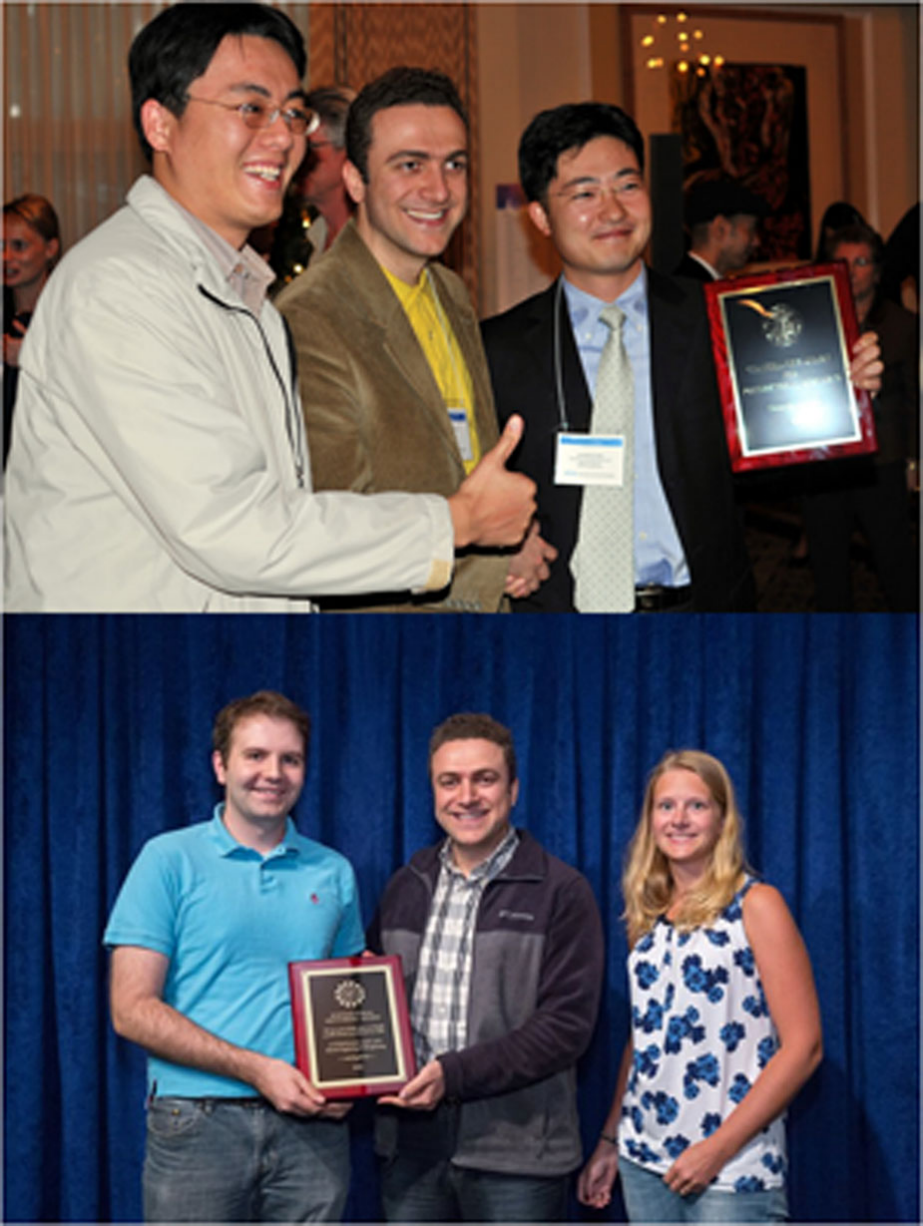
Ozcan Group former Postdocs Dr. Sungkyu Seo (top) and Dr. Euan McLeod (bottom) won the Chancellors Award for Postdoctoral Research at UCLA. Dr. Seo is currently a Professor at Korea University and Dr. McLeod is an Associate Professor at the College of Optical Sciences, University of Arizona.

**Q11: Rising Stars of Light is an annual international competition initiated by LSA to select the brightest young scientists in optics-related fields. Due to the epidemic, 2020 Rising Stars was moved online. After two fierce and friendly contests totaling 8** **h, it was successfully concluded in the presence of more than 500,000 worldwide viewers. 2021 Rising Stars has set sail, and we are honored to invite you again to be the chairman of this competition. What positive effects do you think such events achieve? If it will promote the international influence of LSA?**

A11: I think Rising Stars awards have already become a very prestigious recognition among junior researchers in optics and photonics related fields. This year’s cohort of applications was amazingly well qualified and it was very difficult for the award committee to make a selection for the next round.

Researchers do not work for awards—we are motivated by our curiosity and our passion for “change”—making the world better and advancing our understanding of the universe through the impact of our science and engineering. Having said this, these awards can sometime motivate researchers to continue in their careers, and help them accelerate their progress, reminding them of the importance of their research and broader impact. In this sense, I find the Rising Stars awards very important and timely.

**Figure:**
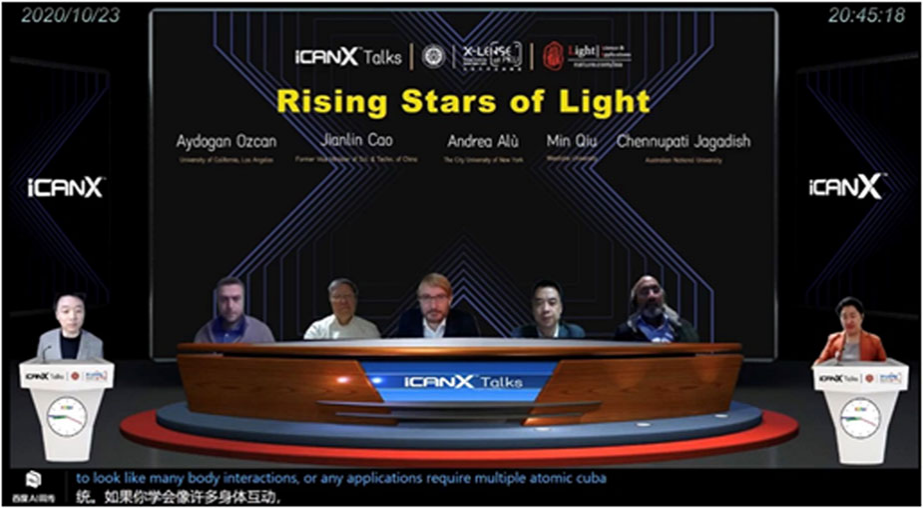
2020 Rising Stars of Light online.

**Q12: In 2017, you were invited by OSA/SPIE Student Branch of Changchun Institute of Optics, Fine Mechanics and Physics (CIOMP) to bring a wonderful OSA Travelling Lecture, which atmosphere on-site was quite warm. What’s your impression of CIOMP? What aspects do you think should be noticed in talent training?**

A12: This was my first time visiting CIOMP. I am very impressed with Optics and Photonics research coming out of China, and over the last 2 decades I have seen Chinese universities in general, together with their students and scholars, making amazing progress. In my lab I have got several generations of Chinese students, from Tsinghua University, Peking University, Zhejiang University and others, and some of my best students are from China. I always eagerly look to see new applications from Chinese universities. The students have an amazing package. They come with an excellent research background, strong publications and are always eager to work hard and learn deeper. Also I personally find Chinese students provide an excellent fit to my lab and its dynamic style—they are very collegial, respectful, and hardworking.

**Figure:**
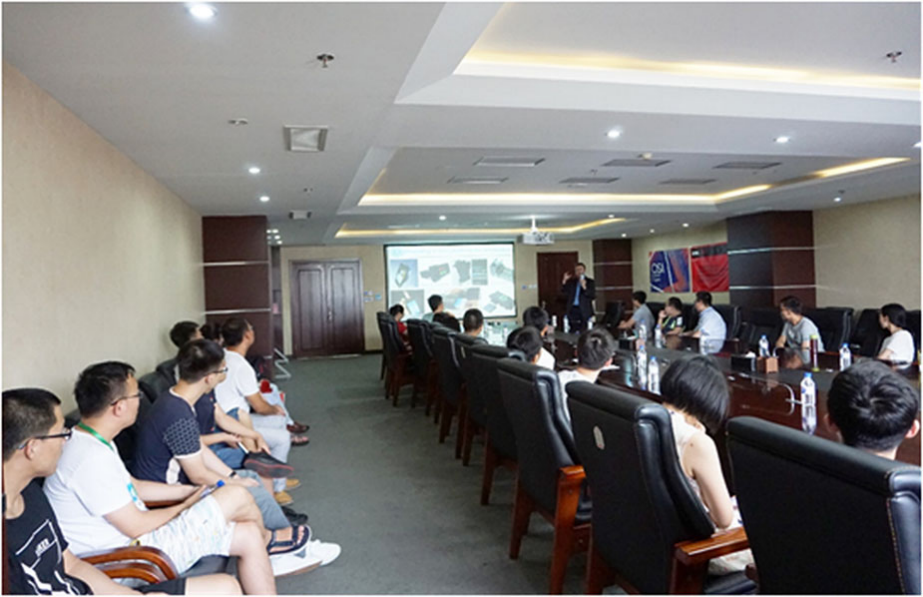
Prof Aydogan Ozcan giving a lecture at CIOMP.

**Figure:**
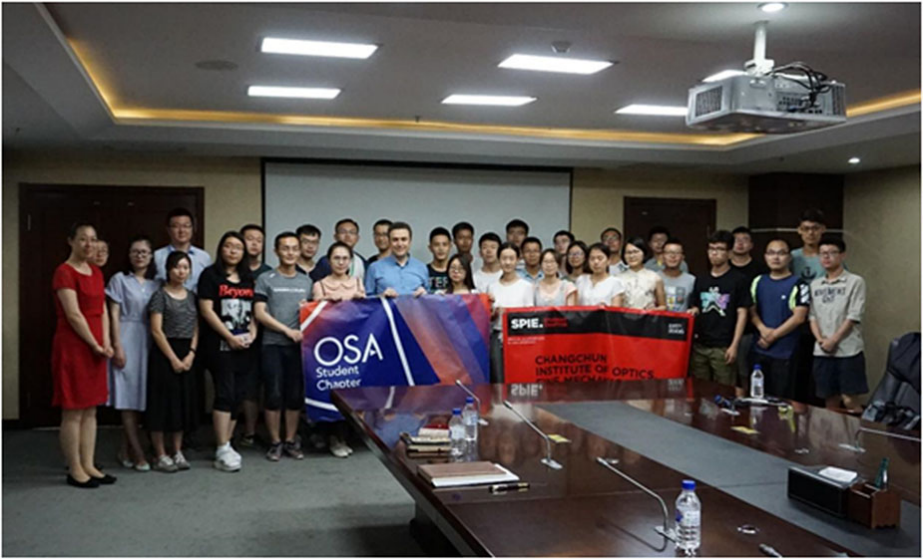
Prof. Aydogan Ozcan with participants at CIOMP.

**Q13: How do you relieve pressure at work? Do you have any interests except for your job?**

A13: I like cooking and inventing new dishes, which mostly end up very tasty (according to my wife), but sometimes perhaps end up with a tasteless invention. I am not afraid of failures in this sense. I also like basketball very much—played at a competitive level until I seriously injured my knees—both of them, one ACL torn after another, one year apart, playing basketball in each case. This also tells something more about me: perseverance and tendency to learn (slowly) through experiments. I stopped playing basketball after losing all the ACL in my knees.

**Figure:**
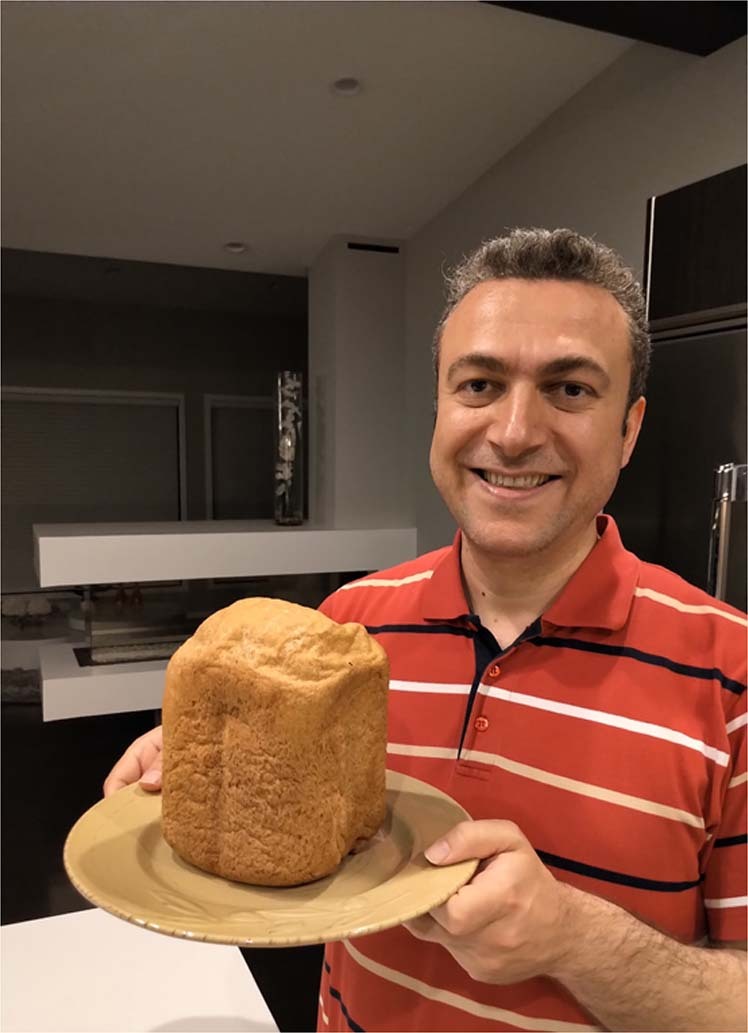
Prof. Ozcan baking bread.

**Light correspondent**

*Tingting Sun is an Assistant Professor of Light Publishing Group at Changchun Institute of Optics, Fine Mechanics and Physics (CIOMP), Chinese Academy of Sciences (CAS). She received Engineering Doctor Degree from University of Chinese Academy of Sciences in 2016. She currently serves as an Academic Editor for an Excellence Program leading journal Light: Science & Applications, and is the Editor-in-Chief Assistant of LSA subclassification journal eLight. She is also a senior talent jointly trained by the International Cooperation Bureau of CAS. She came from the frontline of scientific research and had profound scientific research background. She has presided over two scientific research projects as the project leader, and participated in many major scientific projects. She has published many SCI and EI academic papers and applied for two national invention patents.*

**Figure:**